# Supplementary material for: From in silico prediction to experimental validation: Identification of drugs and novel synergistic combinations that inhibit growth of inflammatory breast cancer cells
Source: bioRxiv. 2025 Dec 16:2025.12.10.693562. Originally published 2025 Dec 13. Preprint. [Version 2] doi: 10.64898/2025.12.10.693562 (PMC12713162; doi:10.64898/2025.12.10.693562)
Supplement: Supplement 1 — S1 Fig. Representative dose response curves for the LWAS drug candidates with efficacy in breast cancer cell lines using the Hoechst assay. (A) SUM159, (B) MDA-MB-231, and (C) MCF-7. Each graph shows the relative cell count (%) plotted against drug concentration (μM) on a logarithmic scale. Plots are grouped by drug mechanisms: antimetabolites, microtubule inhibitors, topoisomerase inhibitors, and tyrosine kinase Inhibitors. Error bars indicate ± SD from replicate experiments. S2 Fig. Representative dose response curves of the GRR effective compounds with efficacy in different breast cancer cell lines using the Hoechst assay. (A) SUM159, (B) MDA-MB-231 and (C) MCF-7s. The x axis represents the concentration of the compound in μM while the y axis represents the cell count % normalized to DMSO control. All the experiments were done in technical and independent experiments (n=3). Error bars indicate ± SD from replicate experiments. S3 Fig. Drug sensitivity heatmap profiles and dose response curves for LWAS drugs in IBC and non-IBC breast cancer cell line panel by MTT assay. (A) Drug response heatmaps across all the breast cancer cell lines. The x-axis represents the concentration of each drug delivered in a dose-response, while the y-axis lists the compounds. The color gradient indicates the level of inhibition, with red indicating higher inhibition and green indicating lower inhibition. Representative dose response curves grouped by drug class: antimetabolites, microtubule inhibitors, topoisomerase inhibitors, and tyrosine kinase inhibitors (TKIs), for (B) SUM149, (C) SUM159, (D) MDA-MB-231 and (E) MCF-7. Inhibition data plotted as the percentage (%) of cell proliferation relative to control versus drug concentration (μM). Error bars indicate ± SD from replicate experiments. S4 Fig. Drug sensitivity heatmap profiles and dose response curves for GRR drugs in IBC and non-IBC breast cancer cell line panel by MTT assay. (A) Drug response heatmaps across all the breast can [file media-1.pdf]

Figure S1

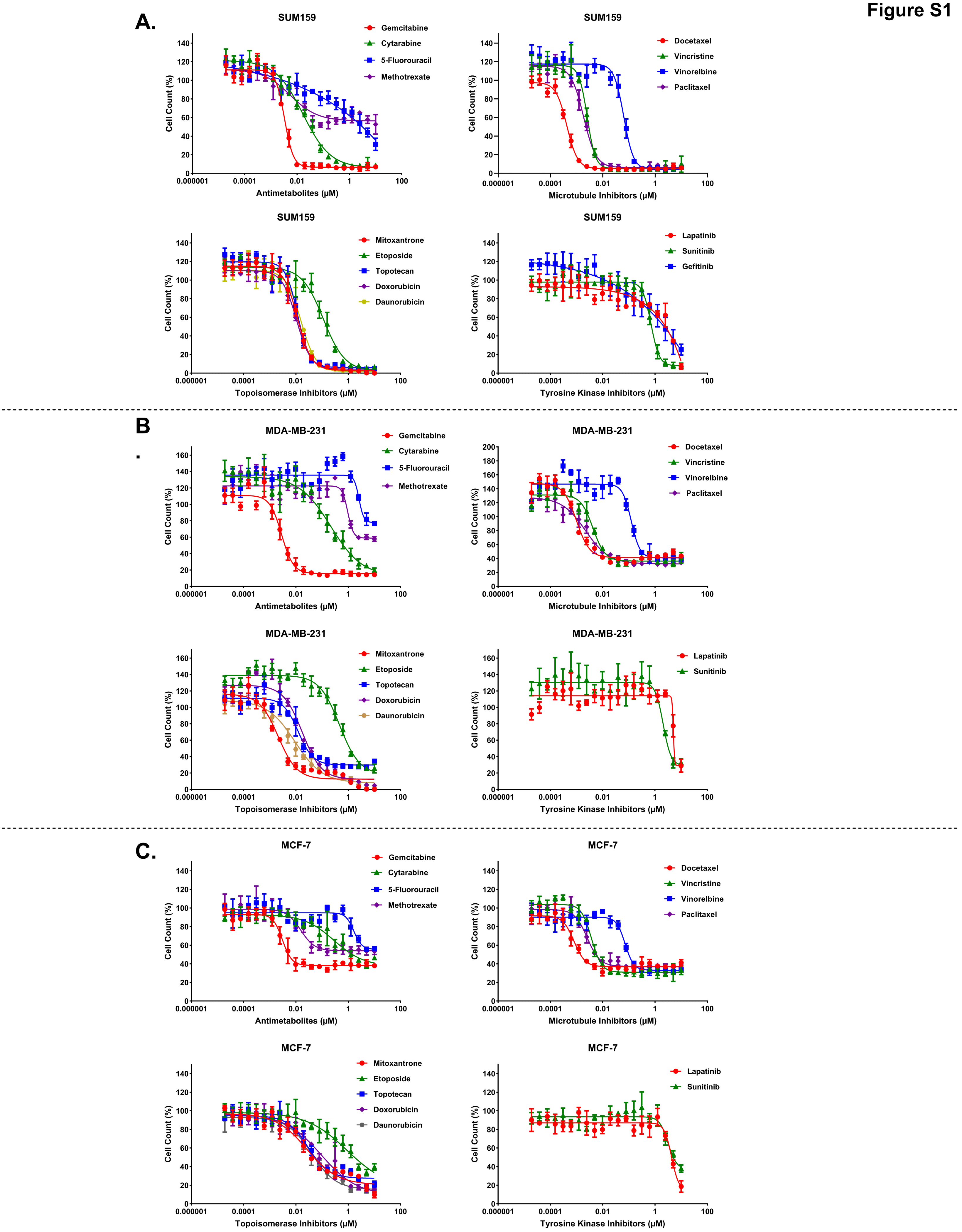

A.
SUM159
Gemcitabine
Cytarabine
5-Fluorouracil
Methotrexate
Cell Count (%)
Antimetabolites (μM)
SUM159
Docetaxel
Vincristine
Vinorelbine
Paclitaxel
Microtubule Inhibitors (μM)
SUM159
Mitoxantrone
Etoposide
Topotecan
Doxorubicin
Daunorubicin
Topoisomerase Inhibitors (μM)
SUM159
Lapatinib
Sunitinib
Gefitinib
Tyrosine Kinase Inhibitors (μM)
B
MDA-MB-231
Gemcitabine
Cytarabine
5-Fluorouracil
Methotrexate
Antimetabolites (μM)
MDA-MB-231
Docetaxel
Vincristine
Vinorelbine
Paclitaxel
Microtubule Inhibitors (μM)
MDA-MB-231
Mitoxantrone
Etoposide
Topotecan
Doxorubicin
Daunorubicin
Topoisomerase Inhibitors (μM)
MDA-MB-231
Lapatinib
Sunitinib
Tyrosine Kinase Inhibitors (μM)
C.
MCF-7
Gemcitabine
Cytarabine
5-Fluorouracil
Methotrexate
Antimetabolites (μM)
MCF-7
Docetaxel
Vincristine
Vinorelbine
Paclitaxel
Microtubule Inhibitors (μM)
MCF-7
Mitoxantrone
Etoposide
Topotecan
Doxorubicin
Daunorubicin
Topoisomerase Inhibitors (μM)
MCF-7
Lapatinib
Sunitinib
Tyrosine Kinase Inhibitors (μM)
0.000001
0.0001
0.01
1
100

Figure S2

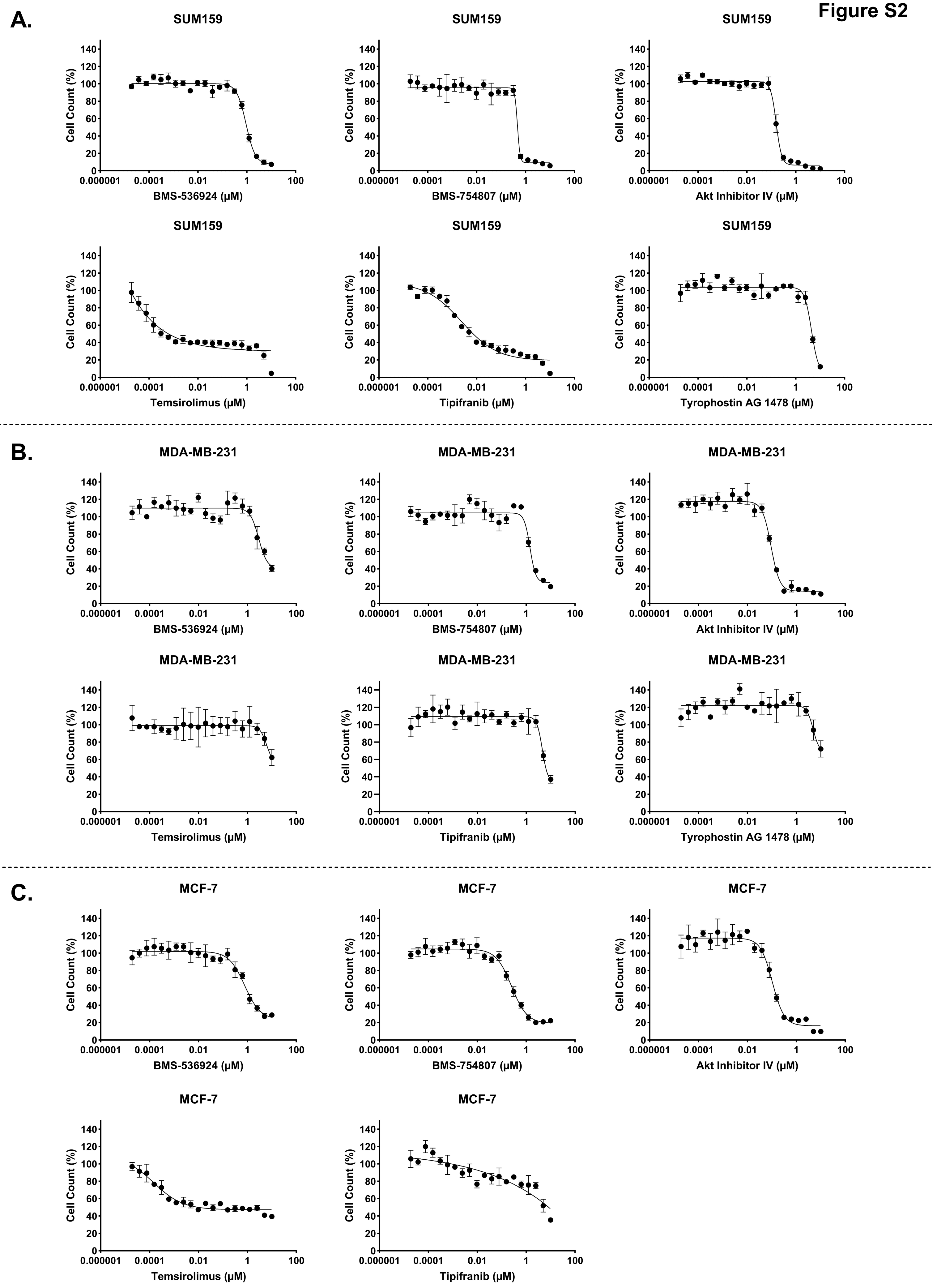

A.
SUM159
Cell Count (%)
BMS-536924 (µM)
BMS-754807 (µM)
Akt Inhibitor IV (µM)
Temsirolimus (µM)
Tipifranib (µM)
Tyrophostin AG 1478 (µM)
B.
MDA-MB-231
Cell Count (%)
BMS-536924 (µM)
BMS-754807 (µM)
Akt Inhibitor IV (µM)
Temsirolimus (µM)
Tipifranib (µM)
Tyrophostin AG 1478 (µM)
C.
MCF-7
Cell Count (%)
BMS-536924 (µM)
BMS-754807 (µM)
Akt Inhibitor IV (µM)
Temsirolimus (µM)
Tipifranib (µM)
0.000001
0.0001
0.01
1
100
0
20
40
60
80
100
120
140

Figure S3A,B

A.

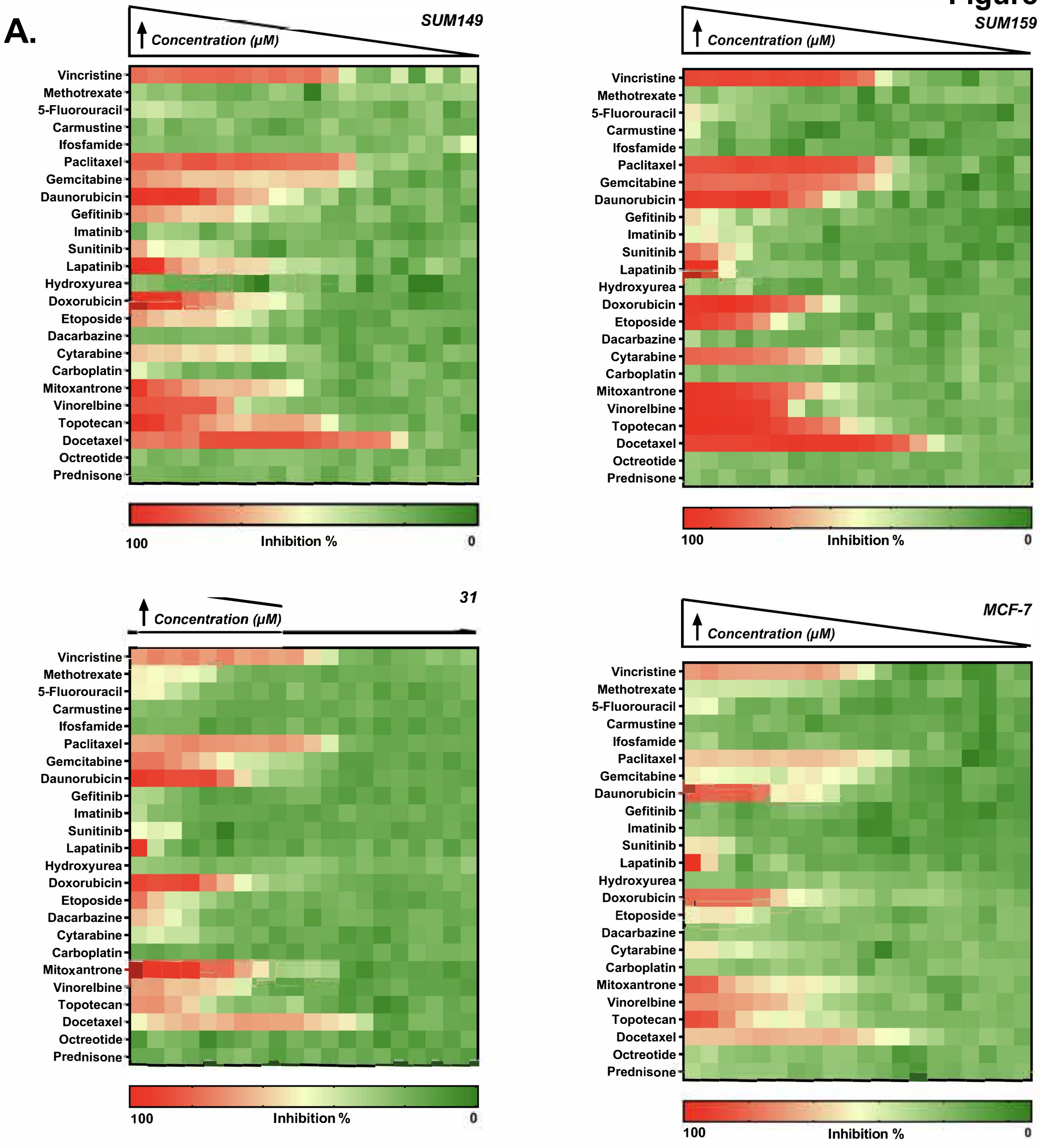

B.

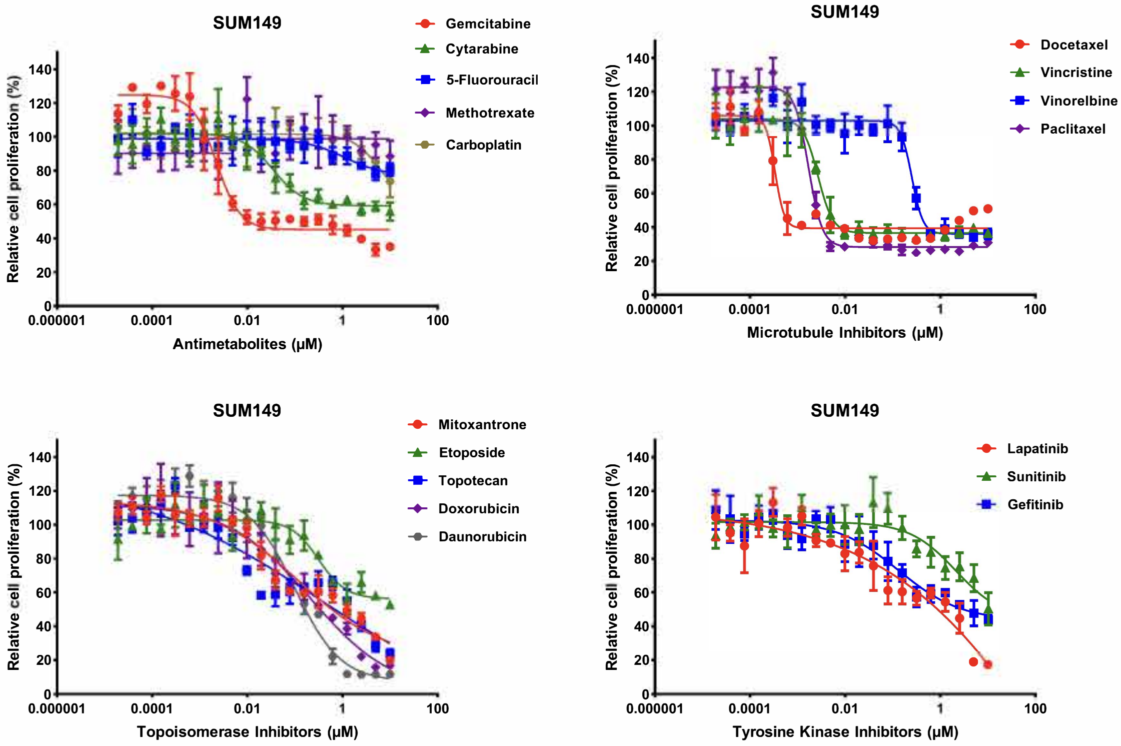

Figure S3C-E

C.

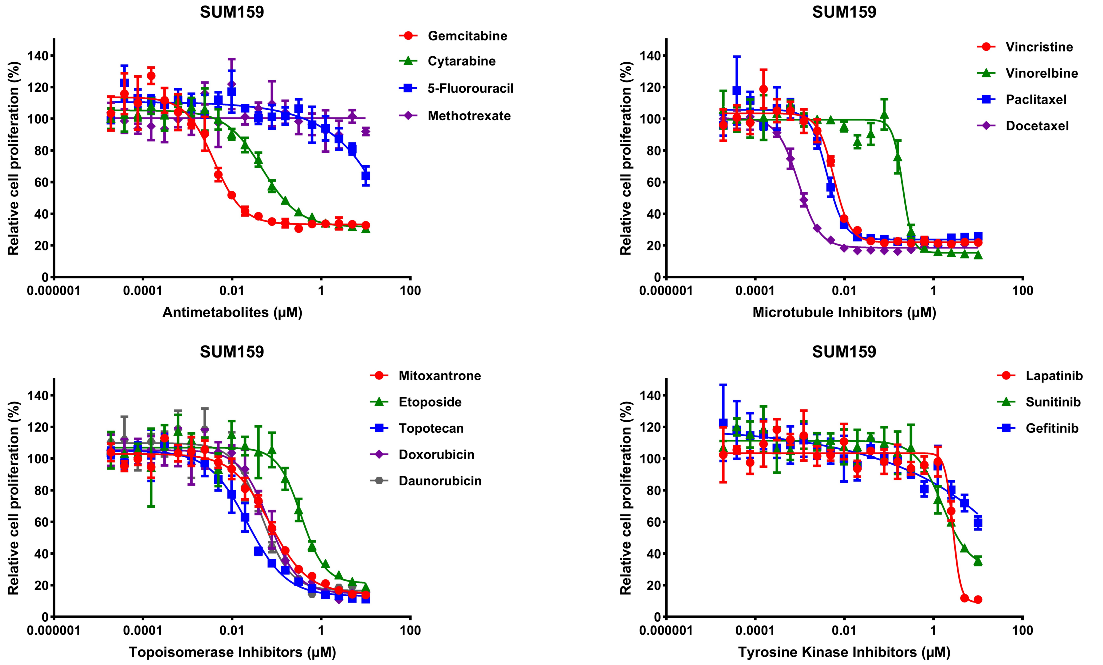

D.

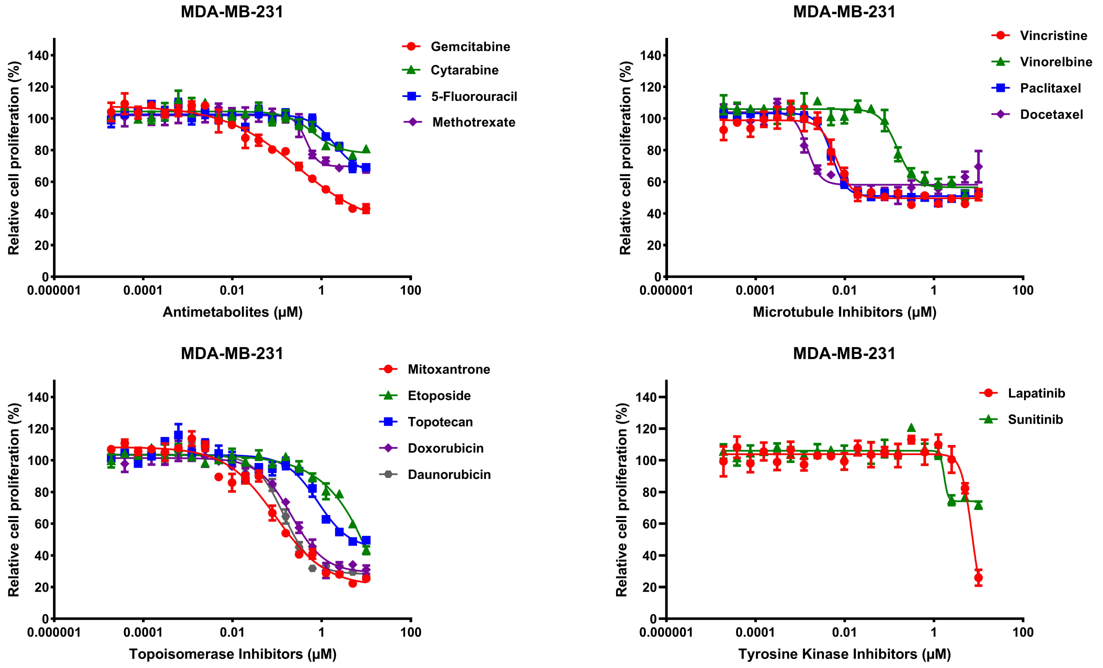

E.

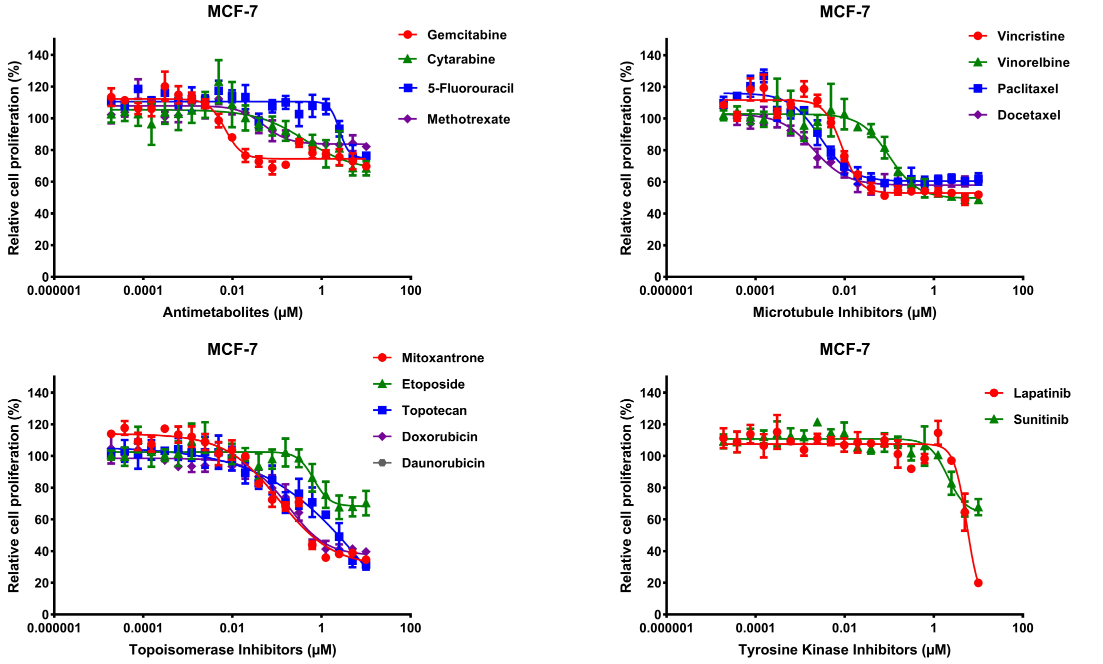

Figure S4A,B

A.

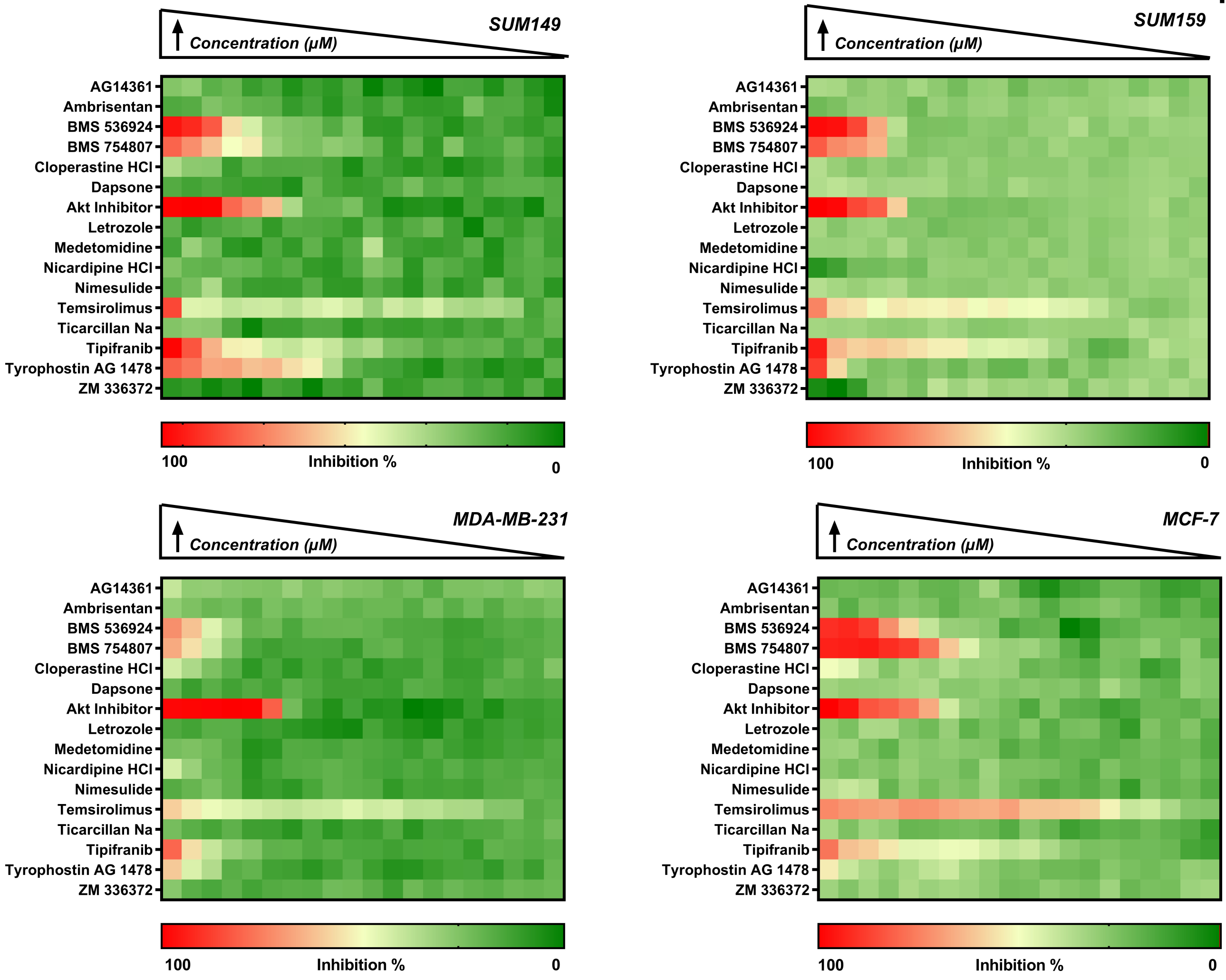

B.

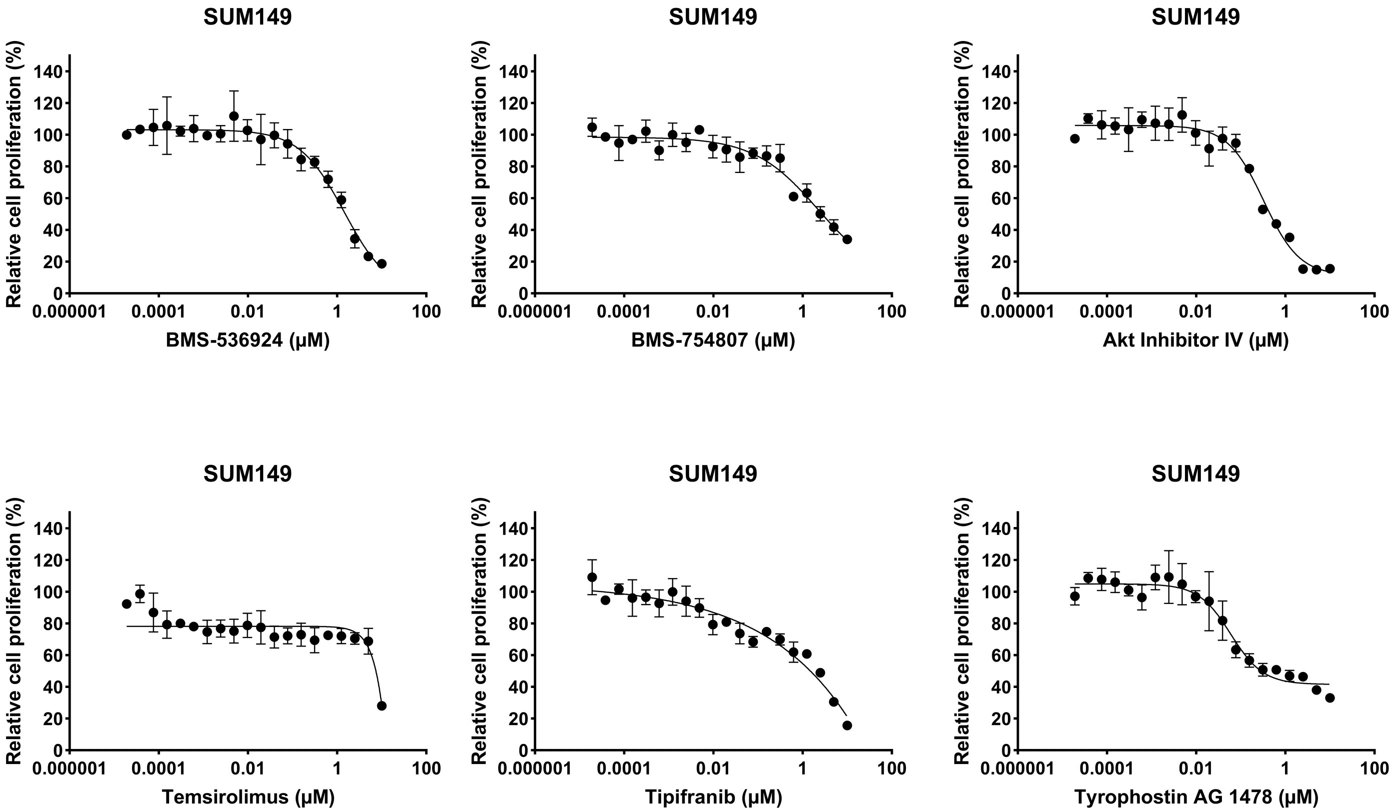

Figure S4C-E

C.

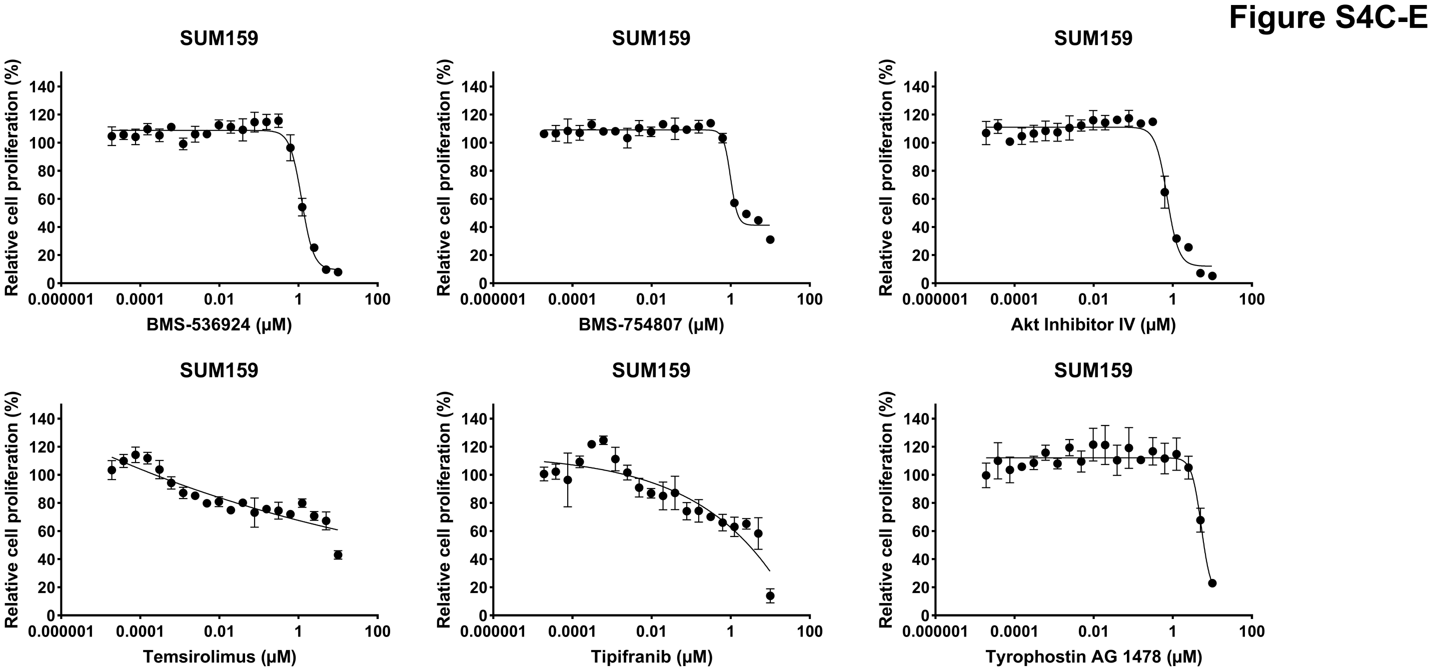

D.

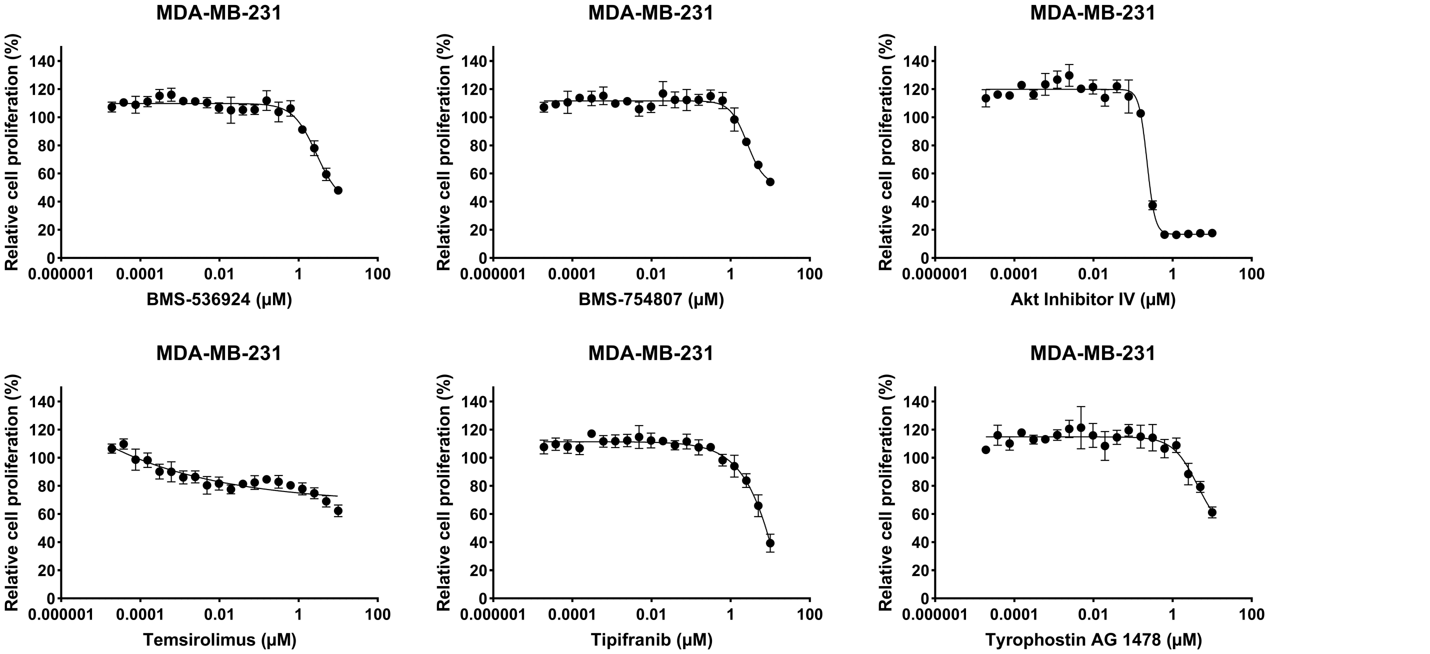

E.

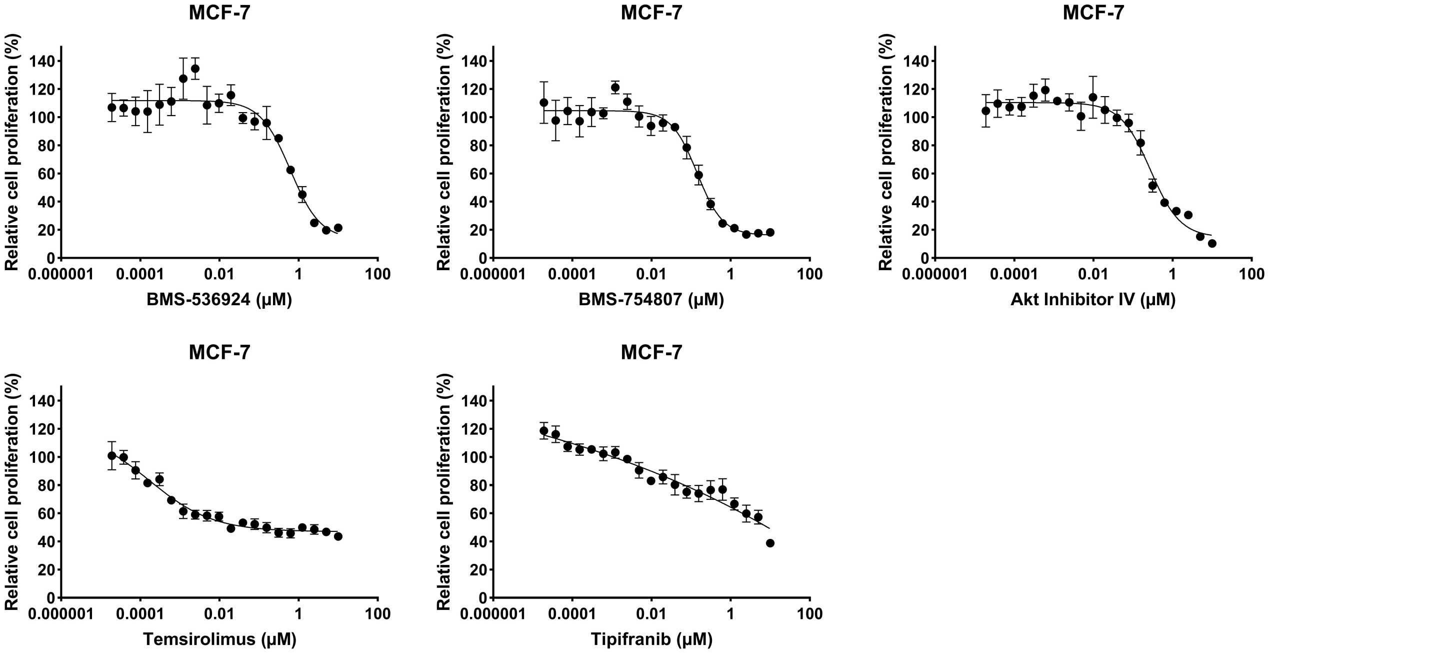

Figure S5A-E

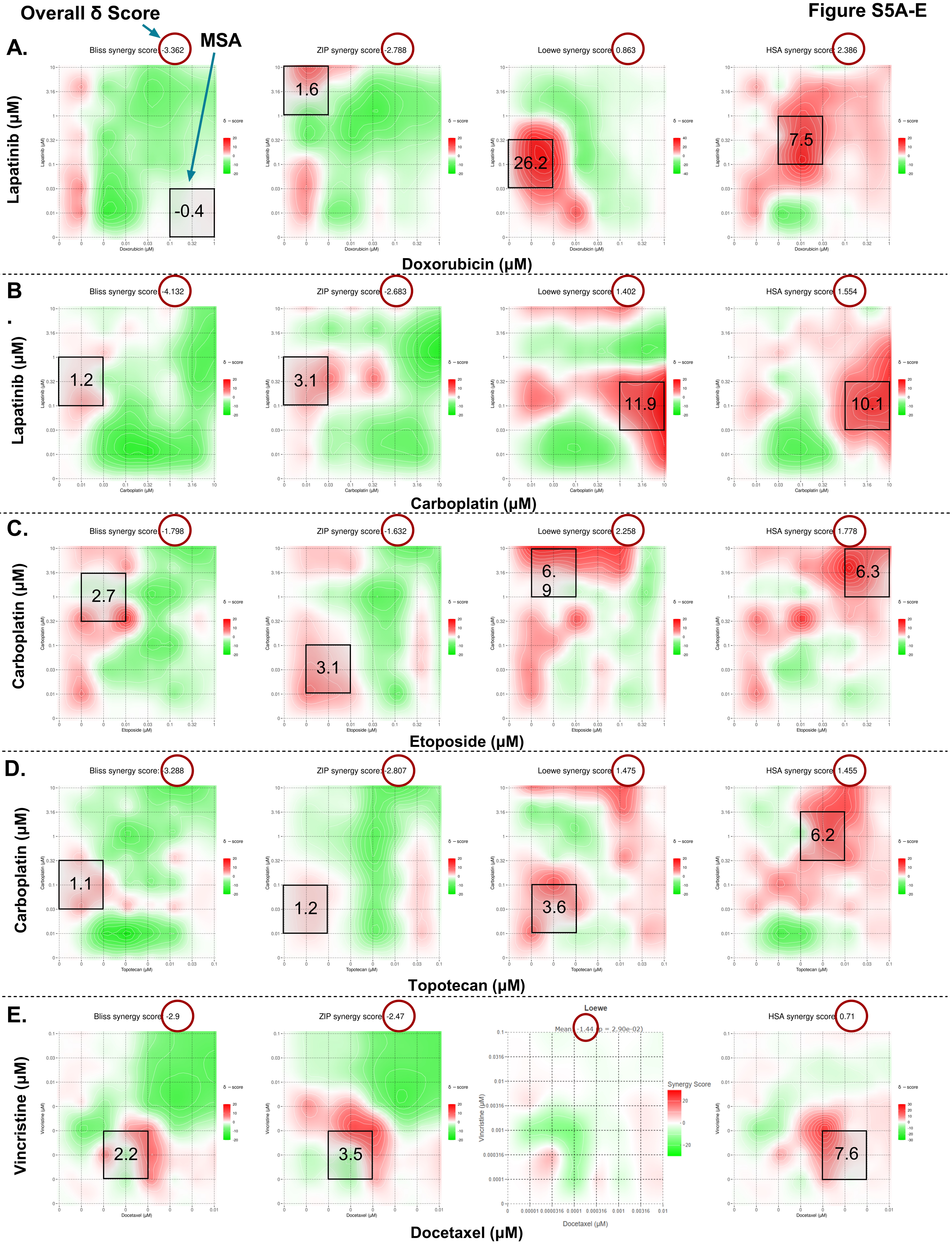

Overall δ Score
MSA
A.
Bliss synergy score: -3.362
-0.4
ZIP synergy score: -2.788
1.6
Loewe synergy score: 0.863
26.2
HSA synergy score: 2.386
7.5
Lapatinib (µM)
Doxorubicin (µM)
B
Bliss synergy score: -4.132
1.2
ZIP synergy score: -2.683
3.1
Loewe synergy score: 1.402
11.9
HSA synergy score: 1.554
10.1
Lapatinib (µM)
Carboplatin (µM)
C.
Bliss synergy score: -1.798
2.7
ZIP synergy score: -1.632
3.1
Loewe synergy score: 2.258
6.9
HSA synergy score: 1.778
6.3
Carboplatin (µM)
Etoposide (µM)
D.
Bliss synergy score: -3.288
1.1
ZIP synergy score: -2.807
1.2
Loewe synergy score: 1.475
3.6
HSA synergy score: 1.455
6.2
Carboplatin (µM)
Topotecan (µM)
E.
Bliss synergy score: -2.9
2.2
ZIP synergy score: -2.47
3.5
Loewe
Mean: -1.44 (p = 2.90e-02)
Synergy Score
HSA synergy score: 0.71
7.6
Vincristine (µM)
Docetaxel (µM)

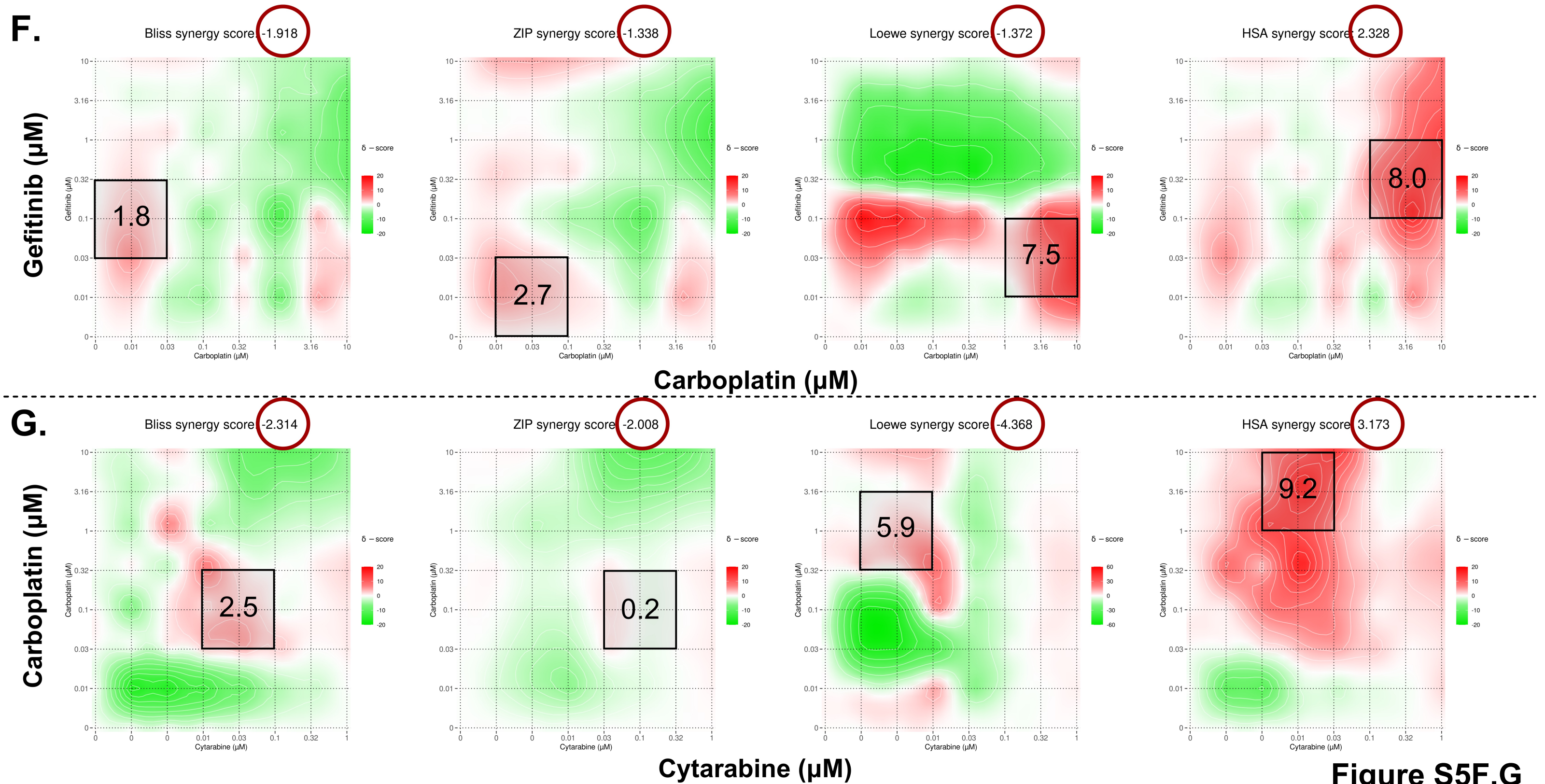
F.
Bliss synergy score: -1.918
ZIP synergy score: -1.338
Loewe synergy score: -1.372
HSA synergy score: 2.328
Gefitinib (µM)
1.8
2.7
7.5
8.0
Carboplatin (µM)
G.
Bliss synergy score: -2.314
ZIP synergy score: -2.008
Loewe synergy score: -4.368
HSA synergy score: 3.173
Carboplatin (µM)
2.5
0.2
5.9
9.2
Cytarabine (µM)
δ – score

Figure S5F,G

Figure S6A-C

A.

2D

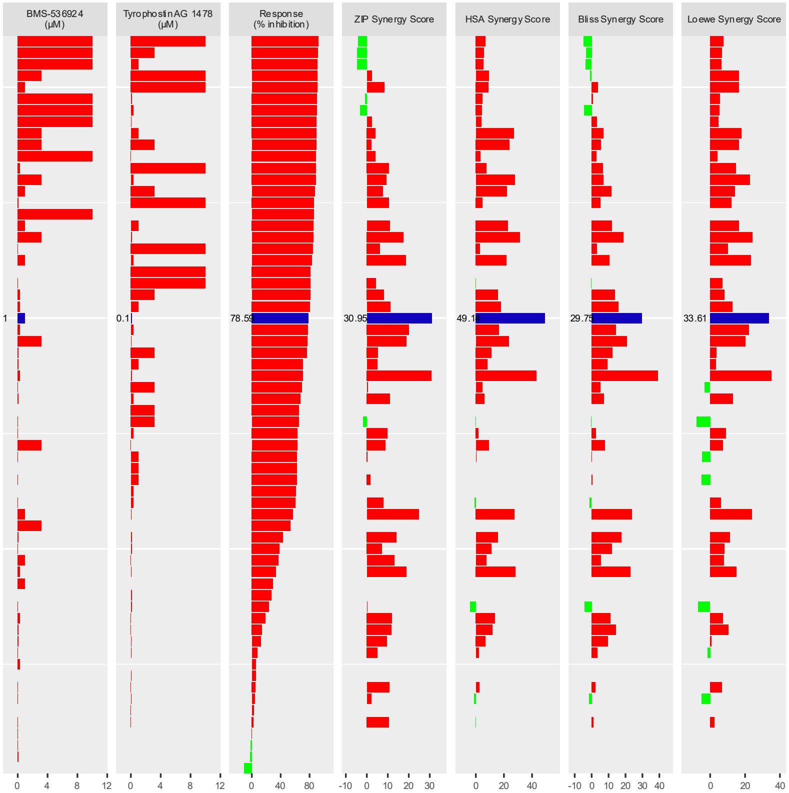

3D

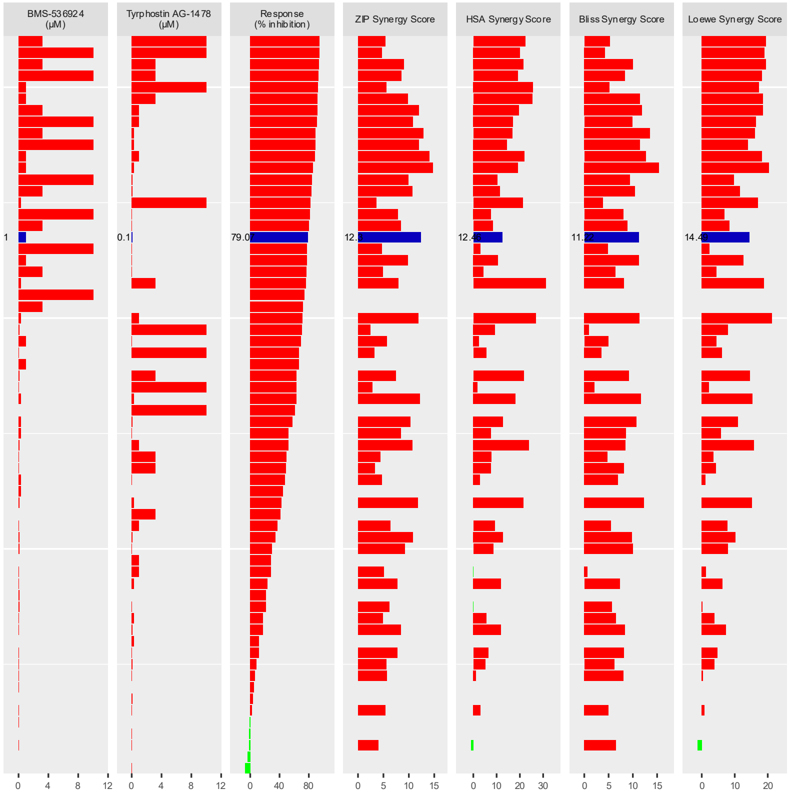

B.

2D

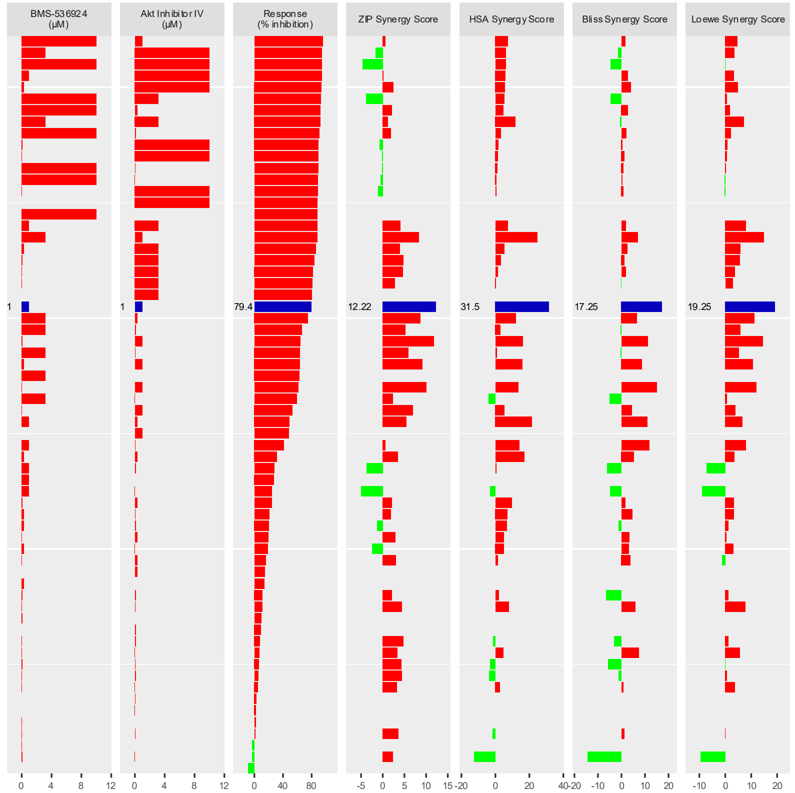

BMS-536924 (µM)
Akt Inhibitor IV (µM)
Response (% inhibition)
ZIP Synergy Score
HSA Synergy Score
Bliss Synergy Score
Loewe Synergy Score
1
1
79.4
12.22
31.5
17.25
19.25
0 4 8 12
0 4 8 12
0 40 80
-5 0 5 10 15
-20 0 20 40
-20 -10 0 10 20
-10 0 10 20

3D

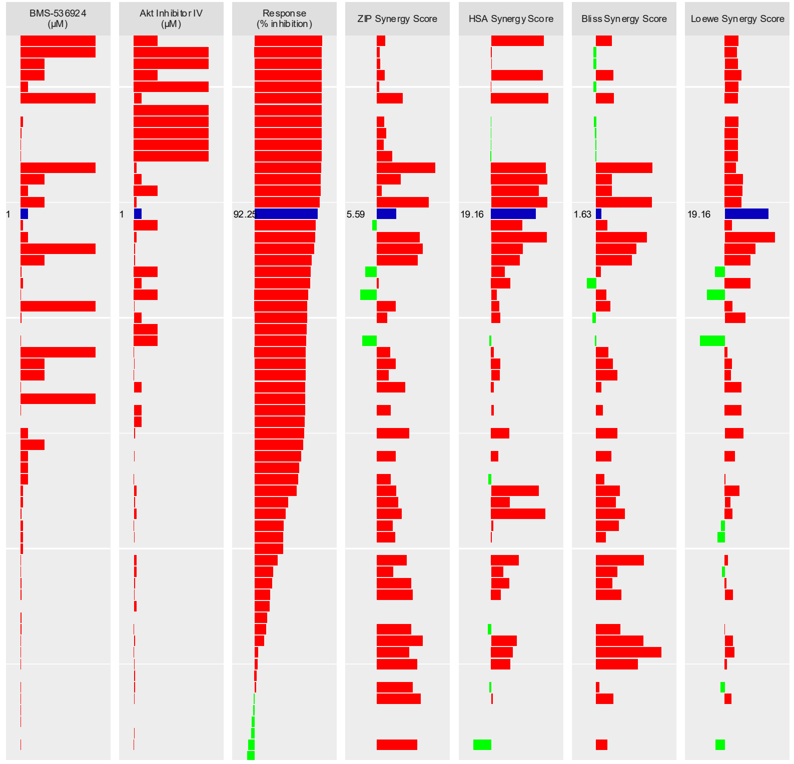

BMS-536924 (µM)
Akt Inhibitor IV (µM)
Response (% inhibition)
ZIP Synergy Score
HSA Synergy Score
Bliss Synergy Score
Loewe Synergy Score
1
1
92.25
5.59
19.16
1.63
19.16

C.

2D

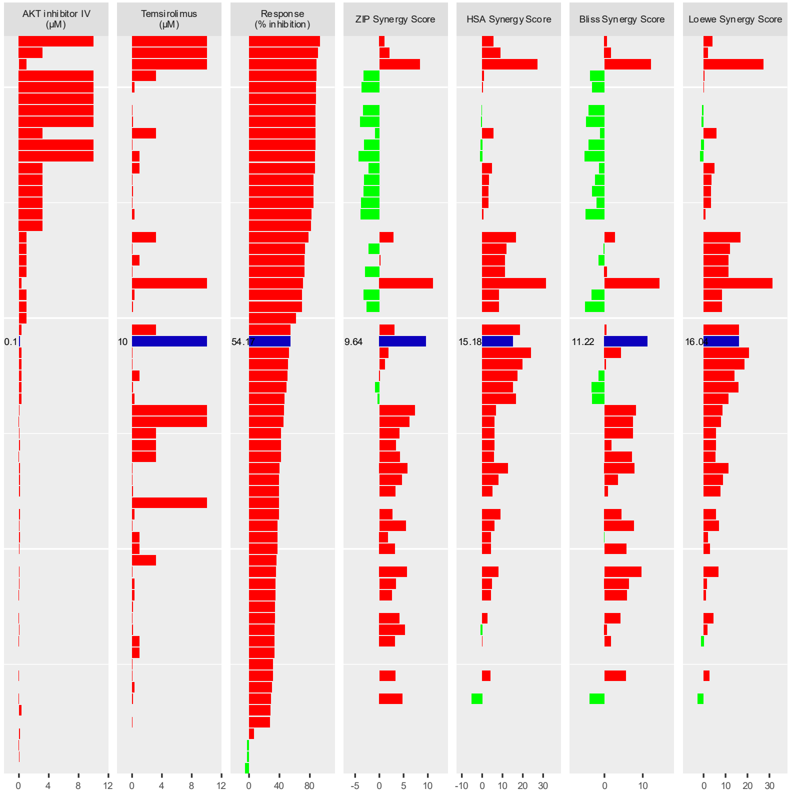
AKT inhibitor IV (µM)
Temsirolimus (µM)
Response (% inhibition)
ZIP Synergy Score
HSA Synergy Score
Bliss Synergy Score
Loewe Synergy Score
0.1
10
54.1
9.64
15.18
11.22
16.08
0 4 8 12
0 4 8 12
0 40 80
-5 0 5 10
-10 0 10 20 30
0 10
0 10 20 30

3D

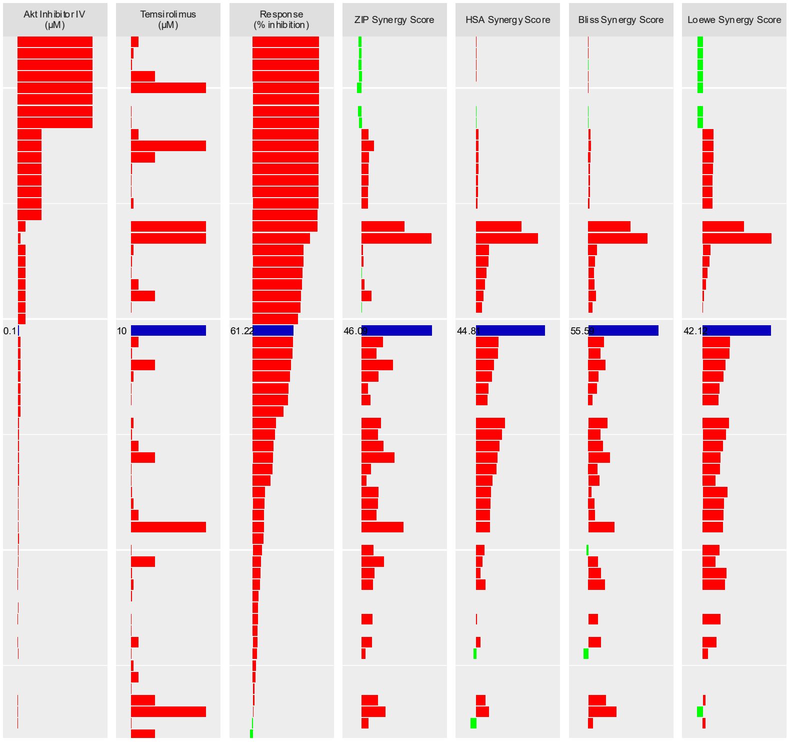
Akt Inhibitor IV (µM)
Temsirolimus (µM)
Response (% inhibition)
ZIP Synergy Score
HSA Synergy Score
Bliss Synergy Score
Loewe Synergy Score
0.1
10
61.22

Figure S7

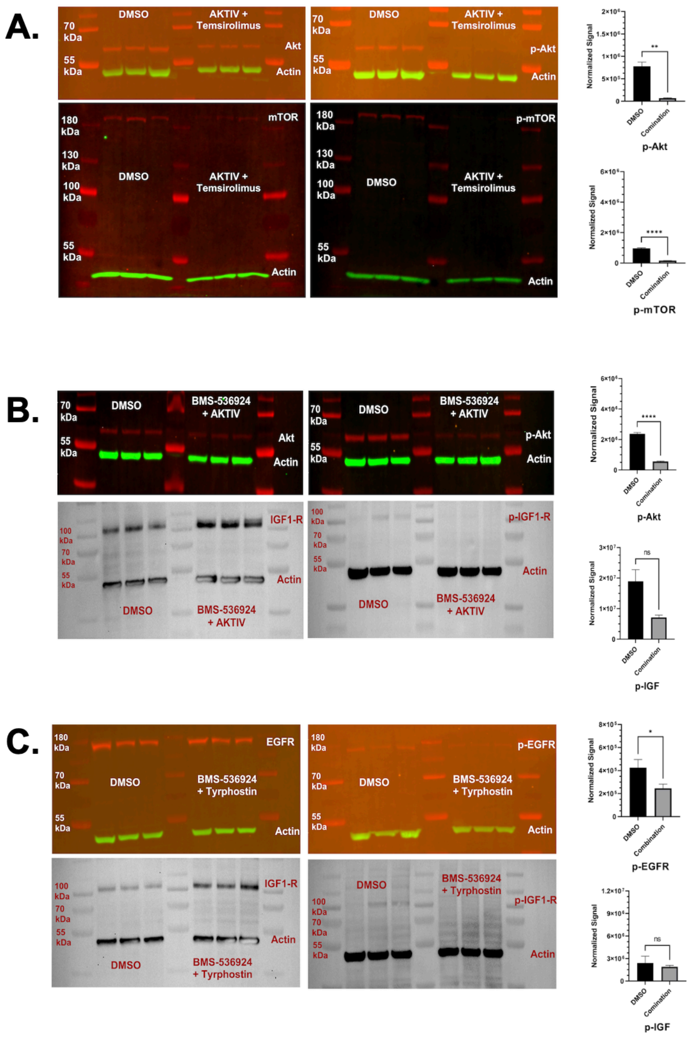
A.
DMSO
AKTIV + Temsirolimus
70 kDa
55 kDa
Akt
Actin
p-Akt
mTOR
p-mTOR
180 kDa
130 kDa
100 kDa
Normalized Signal
DMSO
Combination
p-Akt
p-mTOR
B.
BMS-536924 + AKTIV
IGF1-R
p-IGF1-R
p-IGF
C.
EGFR
p-EGFR
BMS-536924 + Tyrphostin
ns

Figure S8

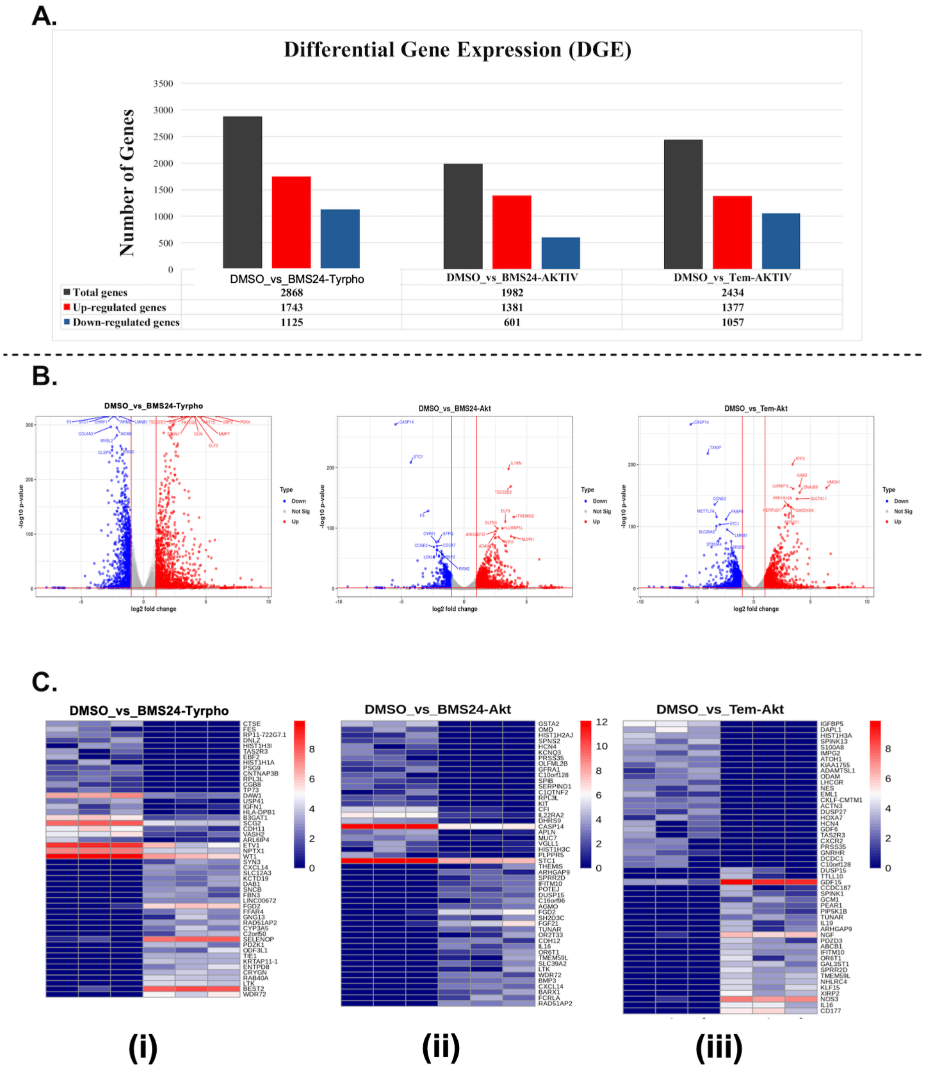
A.
Differential Gene Expression (DGE)
Number of Genes
3500
3000
2500
2000
1500
1000
500
0
DMSO_vs_BMS24-Tyrpho
DMSO_vs_BMS24-AKTIV
DMSO_vs_Tem-AKTIV
Total genes 2868 1982 2434
Up-regulated genes 1743 1381 1377
Down-regulated genes 1125 601 1057
B.
DMSO_vs_BMS24-Tyrpho
DMSO_vs_BMS24-Akt
DMSO_vs_Tem-Akt
-log10 p-value
log2 fold change
Type
Down
Not Sig
Up
C.
DMSO_vs_BMS24-Tyrpho
DMSO_vs_BMS24-Akt
DMSO_vs_Tem-Akt
(i)
(ii)
(iii)

Supporting Information

S1 Table. The 24 LWAS-predicted drugs for IBC (from Ji *et al.*, 2020). The table presents the corresponding drug class, PubMed entry, Clinical Trials entry, and NCT number.

	Drug Name	Drug Bank ID [1]	Drug class (MOA) [2]	PubMed for IBC [3]	Clinical Trials for IBC [4]	NCT Number [5]
**Vinca alkaloids**	Vincristine	DB00541	Microtubule destabilizer	Yes	No	--
	Vinorelbine	DB00361	Microtubule destabilizer	Yes	Yes	NCT01325428
**Antimetabolite**	Methotrexate	DB00563	DNA/RNA damage/inhibits dihydrofolate reductase (DHFR)	Yes	Yes	NCT00003680
	5-Fluorouracil	DB00544	DNA/RNA damage/inhibits thymidylate synthase (TS)	Yes	Yes	NCT01036087<br>NCT00976989<br>NCT02132949
	Gemcitabine	DB00441	Inhibits DNA synthesis	Yes	Yes	NCT00193050<br>NCT00193206
	Hydroxyurea	DB01005	Inhibits DNA synthesis	No	No	--
	Cytarabine	DB00987	DNA replication inhibitor	No	No	--
**Alkylating Agents**	Carmustine	DB00262	Alkylates DNA	Yes	No	--
	Ifosfamide	DB01181	Alkylates DNA	Yes	No	--
	Dacarbazine	DB00851	Alkylates DNA	No	No	--
	Carboplatin	DB00958	Platinum-based alkylating agent (DNA crosslinker)	Yes	Yes	NCT01036087<br>NCT00118053<br>NCT05093387<br>NCT00251329
**Anthracycline**	Daunorubicin	DB00694	Intercalates DNA/ topoisomerase (topo) 2 inhibitor/DNA breakage	Yes	No	--
	Doxorubicin	DB00997	Intercalates DNA/ topo 2 inhibitor/DNA breakage	Yes	Yes	NCT00004925<br>NCT00005822<br>NCT00016406<br>NCT00005800
**Topoisomerase inhibitor**	Etoposide	DB00773	Plant alkaloids (topo 2 inhibitor)	Yes	Yes	NCT00001507
	Mitoxantrone	DB01204	(Intercalates DNA/DNA damage/topo inhibitor)	Yes	No	--
	Topotecan	DB01030	Topoisomerase 1 inhibitor	No	No	--
**Taxane**	Paclitaxel	DB01229	Tubulin stabilizer/mitotic inhibitor	Yes	Yes	NCT00111787<br>NCT01036087<br>NCT00001507<br>NCT02132949
	Docetaxel	DB01248	Tubulin stabilizer/mitotic inhibitor	Yes	Yes	NCT00193050<br>NCT00066443<br>NCT00118053<br>NCT00017095

**Kinase inhibitor**	Gefitinib	DB00317	EGFR Tyrosine Kinase (TK) inhibitor	Yes	No	--
	Imatinib	DB00619	TK inhibitor (BCR-ABL, KIT, PDGF-R)	Yes	No	--
	Sunitinib	DB01268	TK inhibitor (VEGFR/PDGFR/c-kit)	No	Yes	NCT00513695
	Lapatinib	DB01259	TK inhibitor (EGFR/HER2)	Yes	Yes	NCT00105950<br>NCT00558103<br>NCT00111787<br>NCT00450892
**Peptide drug**	Octreotide	DB00104	For metastatic carcinoid tumors and vasoactive intestinal peptide secreting tumors	Yes	No	--
**Glucocort-icoids**	Prednisone	DB00635	Inhibit NF-Kappa B and other inflammatory transcription factors	Yes	No	--

[1] Drug Bank ID; [2] Mechanism of action (MOA); [3] Status in PubMed for IBC; [4] Clinical Trials for IBC; [5] National Clinical Trial Identifier Number.

S2 Table. The 19 GRR-predicted drugs/compounds (from Ji et al., 2023). The table shows with the corresponding drug class for each drug and its mechanism of action.

Drug Name	Drug ID (PubChem) [1]	Drug Class [2]
**AG-14361**	9840076	DNA damage, anti-cancer → PARP-1 inhibitor
**AKTIV**	5719375	Cytotoxic and antiproliferative → Akt protein kinase Inhibitor
**Ambrisentan**	6918493	Vasodilator Endothelin → Receptor, GPCR & G Protein
**AZD-7545**	16741245	Adenocarcinoma → Selective inhibitor of PDHK2
**BMS-536924**	135440466	Anti-cancer → IGF1-R kinase and IR inhibitor
**BMS-754807**	24785538	Anti-cancer → reversible IGF1-R/IR inhibitor
**Butalbital**	2481	CNS depressant → JAK/STAT signaling, P53 signaling, and NOTCH signaling pathway
**Clobenpropit**	2790	Anti-tumor → Histamine H3 receptor, PI3K/AKT pathway
**COT-10b**		Acute myeloid leukemia → Serine/threonine MAP3 kinase
**Dapsone**	2955	Antibacterial → Sulfone drug<br>Anti-inflammatory → (Not fully understood)
**Letrozole**	3902	Aromatase Inhibitor<br>Antineoplastic Agent
**Medetomidine**	68602	Neurological Disease/Psychotic Disorders → selective α2-adrenoceptor agonist
**Nicardipine**	4474	Cardiovascular Disease → Calcium channel blocker
**Nimesulide**	4495	Selective COX-2 inhibitor (NSAID)
**Temsirolimus**	6918289	Antineoplastic (mTOR inhibitor) Immunomodulating agent
**Ticarcillin**	36921	Infection → Beta lactam antibiotic
**Tipifarnib-P2**	159324	Antineoplastic → Farnesyltransferase inhibitors
**Tyrphostin-AG-1478**	2051	Histiocytic Lymphoma → EGFR Tyrosine Kinase Inhibitor
**ZM336372**	5730	Histiocytic Lymphoma → Inhibitor of the MAP protein kinase c-Raf, JAK/STAT signaling.

[1]PubChemID, [2]Drug Class.

S3 Table. Efficacy of LWAS and GRR predicted drugs and compounds in four breast cancer cell lines using the MTT assay. The table lists the IC_{50} values determined for each drug from the MTT assay across the cell lines, with values reported as the mean ± SD from 2 independent experiments.

Study	Drug	SUM149 (IC_{50} μM)	SUM159 (IC_{50} μM)	MDA-MB-231 (IC_{50} μM)	MCF-7 (IC_{50} μM)
**LWAS** [1]	Docetaxel	0.0003 ± 2.3E-05	0.0009	0.002 ± 0.0009	0.002
	Paclitaxel	0.002 ± 0.0001	0.004 ± 0.0008	0.007 ± 0.002	0.003
	Vincristine	0.003 ± 0.001	0.005 ± 0.0009	0.004 ± 0.003	0.009
	Vinorelbine	0.2 ± 0.4	0.2	0.2 ± 0.03	0.1
	Lapatinib	0.7 ± 0.5	3.6 ± 1.2	7.3	5.6
	Sunitinib	2.1 ± 0.05	1.8 ± 0.3	2.1 ± 0.6	2.04
	Gefitinib	0.1 ± 0.02	11 ± 1.9	--	--
	Doxorubicin	0.3 ± 0.1	0.07	0.3 ± 0.1	0.2
	Daunorubicin	0.09 ± 0.01	0.05 ± 0.003	0.2 ± 0.08	0.1
	Mitoxantrone	0.08 ± 0.04	0.07	0.1 ± 0.05	0.05
	Etoposide	0.2 ± 0.1	0.35	7.4	0.7
	Topotecan	0.2 ± 0.2	0.02	0.8 ± 0.08	0.5
	Gemcitabine	0.002 ± 0.0003	0.004 ± 0.0004	0.3 ± 0.03	0.01
	Cytarabine	0.04 ± 0.004	0.05	0.8 ± 0.2	0.4
	Carboplatin	4.5 ± 2.2	--	--	--
	Methotrexate	0.07 ± 0.09	0.1	0.5 ± 0.1	0.05
	5-Fluorouracil	1.1	15.7 ± 3.8	1.9	2.5
**GRR** [2]	BMS-536924	1.5 ± 0.1	1.2 ± 0.2	6.7	0.8 ± 0.1
	BMS-754807	3.6 ± 0.9	2.3 ± 0.7	8.0	0.2 ± 0.1
	AKTIV	0.6 ± 0.2	0.7 ± 0.2	0.3	0.4 ± 0.1
	Temsirolimus	7.3 ± 1.7	0.0005 ± 0.0005	0.0003	0.0005 ± 0.0004
	Tipifarnib	1.3 ± 0.1	2.1 ± 0.7	6.4	3.8 ± 4.8
	Tyrphostin AG-1478	0.4 ± 0.2	5.7 ± 0.3	10.9	--

[1] Drugs identified by LWAS in Ji et al., 2020; [2] Drugs identified by GRR in Ji et al., 2023.

S4 Table. Drugs selected for combination studies. Selected compounds from LWAS and GRR active lists for combination studies in SUM149, along with their corresponding EC_{25} values and the highest concentration used for the 8 x 8 matrix.

	Compounds	IC_{50} Average "Hoechst Assay" (µM)	EC_{25} (µM)	Highest Conc. in 8 × 8 Matrix (µM)
LWAS	Doxorubicin	0.01	0.004	1
	Mitoxantrone	0.005	--	--
	Etoposide	0.07	0.023	1
	Topotecan	0.008	0.003	0.1
	Docetaxel	0.0004	0.0002	0.01
	Vincristine	0.004	0.002	0.1
	Carboplatin	4.4	1.5	10
	Fluorouracil	4.8	--	--
	Lapatinib	0.63	0.12	10
	Gefitinib	0.4	0.2	10
	Sunitinib	1.4	--	--
	Gemcitabine	0.0012	--	--
	Cytarabine	0.025	0.008	1
GRR	BMS 536924	1.9	0.7	10
	BMS 754807	0.5	0.2	10
	AKTIV	0.22	0.1	10
	Temsirolimus	4.3	1.5	10
	Tipifarnib	0.075	0.03	10
	Tyrphostin AG-1478	0.23	0.1	10
